# A Primary Care Clerkship during the COVID-19 Pandemic, An Educator and Learners' Perspective on Telemedicine, The New Frontier of Clinical education

**DOI:** 10.15694/mep.2021.000031.1

**Published:** 2021-02-02

**Authors:** Jesse Bracamonte, Michelle Winscott, Jennifer Talbott, Andrew Pines

**Affiliations:** 1 Mayo Clinic

**Keywords:** telemedicine, COVID-19 pandemic, family medicine clerkship

## Abstract

This article was migrated. The article was marked as recommended.

The COVID-19 pandemic has disrupted the educational experience within medical education. Telemedicine has rapidly ascended to the forefront with healthcare delivery during the pandemic. We discuss our initial perspective with telemedicine as the sole clinical instructive platform within a family medicine clerkship rotation.

## Introduction: Telemedicine and education

Telehealth has rapidly ascended in the wake of the COVID-19 pandemic. Telehealth care may provide the help with access to care to prevent the spread of a disease or provide care when medical offices close. Medicine and medical education has had to evolve and develop new teaching strategies that place the needs of the patient first, providing high quality care, and properly teaching and instructing students with this growing model of care ( Lurie and Carr, 2018). Although telemedicine is not new, its use has exponentially risen among the COVID-19 pandemic. Prior to the pandemic, Mayo Clinic conducted approximately 30 telemedicine video visits daily. Over the past 3 months, the video visits known as telemedicine has increased to approximately 3,000 video visits daily among many specialties. The video visits as of May have now been used as the only clinical place of instruction given the need for social distancing. A 2017 survey among family physicians reported that a lack of training and experience with telemedicine was one of the barriers to using telemedicine in their practice ( Moore *et al.*, 2017). As medical students rotate through different medical disciplines, it will be important for them to receive adequate training in patient virtual care clinical format. Medical schools are learning to incorporate telemedicine competencies into the medical school curriculum to improve access to care, improve quality of care, and reduce health care expenses ( Waseh and Dicker,2019). As for our experience, due to the pandemic, we were the first students in Mayo Clinic Arizona to be clinically instructed by family medicine physicians throughout our entire clerkship.At the time, there was no available option, other than the telehealth experience.Therefore, this type of distance learning poses a unique challenge: Can we students be effectively educated what is expected to learn from the safety of our own home using a laptop?

## Setting

This May 2020, third year students on the Arizona campus of the Mayo Clinic Alix School of medicine piloted an entirely virtual three week long family medicine clerkship. Each day students worked with a dedicated core of family medicine physicians to connect and conduct video appointments by Zoom to patients within the Mayo Clinic Arizona Family Medicine practice. The experience was new for precepting physicians, patients, and students alike as many were using Zoom for the first time and for all was the first to time to participate in a video visit.

## Telemedicine: the educational experience

For medical students, there were opportunities to facilitate the clinical interview, review medications, and obtain a history and subsequently feel independent. Some preceptors turned off the video modality function of Zoom and listened to the conversation,and rejoining the clinical interaction when the assessment and plan was to be presented. Other appointments were three -way collaboration with the patient, student, and attending physician. Students were provided the opportunity to gather the history, perform a visualized exam pending on the problem, and provide the assessment and plan with the attending being active on the screen. For many patient complaints, students still had the opportunity to examine patients through the screen and effectively guide patients through physical exam maneuvers. Appointments were concluded with the student sharing the assessment and plan and awaiting the final recommendations from the attending physician. The student would then document the note and await the preceptor’s co-signature.

Students were able to conduct detailed and thorough physical exams virtually such as with examining musculoskeletal complaints. Training materials were available regarding common examination techniques which could be facilitated easily within the virtual visit. Video instruction as provided by Stanford health was useful. Such instruction provided informative techniques such as with examining a patient’s throat, evaluation for back pain, and evaluation of the shoulder and upper extremity.

The medical diversity which makes family medicine so special was evident with virtual telemedicine as well. Students were able to evaluate chronic disease management entities such as diabetes, hypertension, asthma, and dyslipidemia and also evaluated acute entities such as upper respiratory infections and cough. The latter allowing for the benefit of providing infectious transmission by allowing the patient to be evaluated from home. Video visits, observably allowed a perceived intimacy in which patients appeared to be more likely to share their symptoms of depression, anxiety or substance use with the care team as noted by the attending physicians. The students noted the importance of longitudinal care with family medicine, as patient’s comfort and interaction with the physicians demonstrated the nurturing and caring relationship within family medicine.

The virtual family medicine clerkship had many positive aspects. Clearly observed benefits included the efficiency of a properly conducted visit, the vast majority of disease entities easily facilitated additional self-directed learning after clinic hours, and the importance of having longitudinal relationships with patients. The students were able to further understand the patient’s home environment and to tailor the care plan based on the clinical home setting, such as with a parent who is simultaneously trying to participate in a video exam while taking care of her toddlers.Downsides for the telehealth medical education experience included less emphasis on physical exam findings and the robust experience with learning specific exam maneuvers and techniques germane to many entities seen in a primary care setting.We also noticed more “sedentary” sitting for the student and preceptor. Ability to maintain one’s awareness of appropriate work ergonomics while working from home became quite evident.

## Final Thoughts on a New Wave of Clinical education

Telemedicine, the new wave of delivery of medicine amongst the COVID-19 pandemic is a venue of medicine to provide care to those while preventing the transmission of virus. The opportunity to be educated in this venue provided many insights with patient care, and provided a convenient way to deliver care in a time and environment that was potentially unsafe to do so otherwise. It is important to not lose sight of what drew us to medicine in the first place; friendly banter and small talk that helps us connect with our patients, a comforting hand on the shoulder, or a hand shake thanking a patient coming into the office. It is those intangible and important aspects that make family medicine special.As telehealth becomes more normalized, it is critical that we don’t lose sight of the power of touch and connection, in ways that a video visit can’t provide-It is our ability to connect with each other and the innate gift of touch after all that makes us human!

## Take Home Messages

• Telemedicine has rapidly become an essential part of healthcare delivery.
• Telemedicine has ascended as an instructional platform as well.
• We must further develop strategies to make this healthcare delivery system the best for both educators and learners

## Notes On Contributors

**Dr. Jesse Bracamonte** is a board certified family medicine physician and is a fellow in the American Academy of Family Physicians. He is an assistant professor in the Mayo Clinic Alix School of medicine in Scottsdale, Arizona.

**Dr. Michelle Winscott** is a board certified family medicine physician. She is the lead director for the family medicine clerkship in the Mayo Clinic school of medicine in Scottsdale, Arizona. She is an assistant professor in family medicine in the Mayo Clinic Alix School of Medicine.

**Ms. Jennifer Talbott** is an MD candidate in the Mayo Clinic Alix School of Medicine in Scottsdale, Arizona. She completed her undergraduate degree at the University of Chicago in Chicago, Illinois. She is also a Master’s of Public Health candidate through Columbia University in New York. She plans to enter an obstetrics/gynecology residency in 2021.

**Mr. Andrew Pines** is an MD candidate in the Mayo Clinic Alix School of medicine in Scottsdale, Arizona. He graduated with a bachelor’s degree from the Univeristy of Denver in Denver, Colorado. He plans to enter a psychiatry residency in 2021.
