# Supplementary material for: A Primary Care Clerkship during the COVID-19 Pandemic, An Educator and Learners' Perspective on Telemedicine, The New Frontier of Clinical education
Source: MedEdPublish (2016). 2021 Feb 2;10:31. [Version 1] doi: 10.15694/mep.2021.000031.1 (PMC10939602; doi:10.15694/mep.2021.000031.1)
Supplement: Peer review history

# Reviewer report: Reviewer response for version 1

**DOI:** 10.21956/mep.18855.r26885

**Published:** 2021-05-06

**Authors:** Ken Masters

**Affiliations:** 1 Sultan Qaboos University

This review has been migrated. The reviewer awarded 3 stars out of 5 An interesting opinion piece from medical educators and learners on telemedicine during the COVID-19 Pandemic.The paper shows the positive impact that telemedicine and telehealth can have. On this point, the paper is very strong.Every new innovation, however, comes with a cost. Sometimes financial, but other costs also. The authors do mention a few “downsides,” but do not really give a complete picture. For a start, in order for the success described in the paper, an enormous organisational and infrastructural system is required: this must be implemented, maintained, and developed. The authors speak of the required training, but do not acknowledge the difficulties of such training. Although the paper is an opinion piece, in order for it to have real value to the readers, it does need to provide balance, otherwise, readers will be left with the impression that one must simply implement telemedicine. (It may be that the authors operate in an environment in which these are not issues of any concern, but then that also needs to be acknowledged, and will, unfortunately, greatly reduce the value of the article to almost all medical educators around the world).So, some useful insights, but, I would like to see Version 2 that balances the optimism with realism.

Reviewer Expertise:

NA

No decision status is available

# Reviewer report: Reviewer response for version 1

**DOI:** 10.21956/mep.18855.r26884

**Published:** 2021-02-02

**Authors:** Johnny Lyon Maris

**Affiliations:** 1 Southampton GP Education Unit

This review has been migrated. The reviewer awarded 4 stars out of 5 Thank you for presenting this descriptive article which talks about the use of video consultation. I think we have to be very careful with semantics, telemedicine is used correctly here, and I think the definitions of telecare and telehealth would be useful in this article. In some regions telemedicine colloquially has been used for the monitoring of patients physiological and perceived wellness and this could cause confusion.That being said having a medical student as an active participant or observational capacity, and the preceptor likewise is an excellent addition to the use of the technology.It is important to have descriptions of where this has worked well for improvements to be adopted.

Reviewer Expertise:

NA

No decision status is available
